# Velocity field and turbulence structure of the meandering flow produced by alternating deflectors

**DOI:** 10.1038/s41598-024-58264-8

**Published:** 2024-04-02

**Authors:** Jie-min Zhan, Wing-hong Onyx Wai, Yu-tian Li, Wen-qing Hu, Ying-ying Luo

**Affiliations:** 1 Department of Applied Mechanics and Engineering, Shenzhen Campus of Sun Yat-sen University, Shenzhen, 518107 China; 2 Department of Civil and Environmental Engineering, The Hong Kong Polytechnic University, Hong Kong, 999077 China

**Keywords:** Deflectors, Meandering flow, Horseshoe vortex system, Large eddy simulation, Civil engineering, Fluid dynamics

## Abstract

Meandering flow can be formed during the advance of natural rivers by the scouring of river banks. However, this phenomenon is not common in artificial cement channels. This study used experimental scouring terrain data for a numerical simulation to study the meandering flow pattern formed between double alternating deflectors in a straight channel. The numerical results showed that the path of the accelerated flow generated by the upstream deflector was changed by installing a downstream deflector while the flow rate remained unchanged. Thus, a meandering flow formed, and a stable, narrow, high-speed zone formed in the downstream area. The accelerated flow between the two deflectors hit the channel bank soon after its direction changed. Then, a strong downward flow formed in this area, which may have produced an elliptical scour hole. A large-scale vortex structure was formed in the elliptical scour hole, which was influenced by the horseshoe vortex system before the downstream deflector.

## Introduction

In recent years, excessive human disturbance has damaged the natural ecosystem of many rivers. For the sake of land conservation and flood discharge safety, people cut the bend of the river and make it straight, so the section of the river is basically unchanged. This kind of straight channel reduces the diversity of the water flow ecological environment and leads to the degradation of the river ecosystem. In order to meet the needs of protecting the environment, ecological restoration should be carried out on these rivers, especially urban rivers, to make them into meandering flow with pool and riffle sequences^1^. At present, the ecological restoration methods of straight rivers include the reconstruction of hydraulic dynamics in a small scope, such as the urban river during the low flow rate period^2^. Considering the limited space and the need for short-term flood discharge of urban rivers, ecological restoration buildings such as deflectors can be built in the channel so as to form a meandering flow pattern during the low flow rate period.

A deflector, shaped like a spur dike, is a commonly used hydraulic structure in river training projects. Its purpose is to protect riverbanks, maintain the channel depth and promote aquatic diversity by deflecting and contracting the incoming flow^3,4^. However, the construction of the spur dike can cause damage to the structure itself due to the complex flow and local scour^5^. Excessive scour should be avoid to maintain stability^6,7^. Therefore, lots of researchers had conducted experimental and numerical studies on the local scour problem near the spur dike^8–11^ to find the main factors which contribute to the sediment transport. In general, there are three main factors that induce local scour^12^: the increase in flow velocity per unit width, the down flow in front of the spur dike and the horseshoe vortex system (HVS). The constriction of the river channel due to the spur dike increases the flow velocity near the head and this increase in velocity result in an increase in the scour capacity by the water flow, which is the direct factor that initiates local scour. Meanwhile, some of the obstructed water was diverted to rush the riverbed, also known as the down flow^13,14^. Unlike typical sediment transport caused by the shear stress between the two layers of water and sand, down flow can stir up the sand layer by direct collision between water and sand. HVS have the potential to enhance the sand transport capacity of the water by creating complex vortex-flow in the near-bed area^15–17^. Due to the higher internal turbulence intensity in the vortex-flow compared to the surroundings, the sediment particles are pushed by the strong turbulent stresses at the bed surface^18–21^.

Local scour is usually a complex phenomenon resulting from a combination of several main flow patterns. As the river bed  deforms, it forced the flow to alter until a dynamic balance has been achieved between the water and the bed^22^. Biron et al.^9^, in their study about the three-dimensional flow structure in the vicinity of the spur dike, emphasized the importance of the understanding of the interaction mechanism between topography and hydrodynamic properties for the research about the local scour problem. However, studies of the evolution of the main flow structures causing scour in response to topographic changes and their temporal effects on sand transport are lacking. Unger and Hager^23^ and Guan et al.^24^ conducted experimental studies on the evolution of the HVS during the development of the scour hole. It was found that the HVS were temporarily dissipated during an early scouring stage of a rapidly changing topography change caused by the down flow deflection. Si et al.^25^ found that during the local scouring process there is a specific stage where the mean turbulent kinetic energy of the main vortex in the flow field is reduced to its minimum value and then gradually recovered as the scour hole grows. It is clear from the above studies that changes in the topography have a significant effect on the original flow field. Before the scour reached equilibrium, the main flow structure may have gone through a phase where the strength or energy of the flow reached its maximum value. Koken and Constantinescu^26,27^ applied numerical techniques to understand the mechanism of sediment transport in the vicinity of an experimental scour hole. The results show that more realistic and accurate flow field data can be obtained by numerical calculations based on the experimental topography. This allows the visualization of the HVS and characterization of their shapes and positions relation to the bed.

Previous studies on deflectors have generally focused on the flow field and vortex structure of a single spur dike, a pair of deflectors, or deflectors in series. The main causes of scour in these cases are accelerated flow and high-energy eddies near the bed region. There have been few reports on the flow structure generated by alternating deflectors, which could induce a meandering flow pattern. In this study, experimental data and large eddy simulation methods were used to investigate the meandering flow characteristics and vortex structures induced by two alternating deflectors.

## Experiment setup

**Figure 1:**
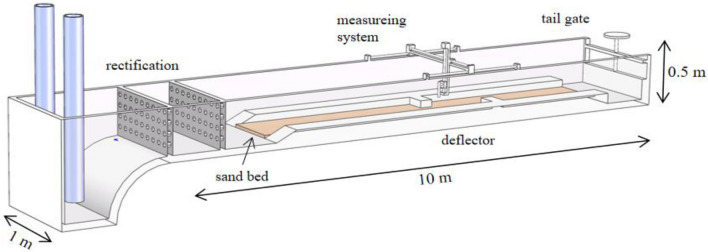
Schematic diagram of experimental flume.

The clear-water scour experiments were carried out in a rectangular tank at the Hong Kong Polytechnic University, as depicted in Fig. 1. The length, width, and depth of the flume are 10 m, 1 m, and 0.5 m, respectively, and the slope of the channel is 0.0043. Because of the small size of the designed experimental model, the width of the tank was reduced from 1 to 0.2 m by inserting a plastic wall into the tank. In order to minimize the wave by dissipating the excess energy at the inlet, a floating pad was placed on the water surface. A manual weir gate was installed at the end of the tank to control the water depth. The experimental local scour section was placed 4 m away from the entrance, which is 1.5 m long, 0.2 m wide, and 0.03 m thick. The sand grains filled in this area are uniform with $d_{50} \approx 0.5\;{\text{mm}}$. The upstream and downstream zones of the test section were filled with non-uniform sand with a diameter ranging from 0.5 to 1.2 mm, and the thickness of the sand layer was also 0.03 m. The first deflector ($0.06\;{\text{m}} \times 0.08\;{\text{m}} \times 0.2\;{\text{m}}$) was placed 5 m away from the entrance. The flow rate is 0.9 L/s in single and double deflector scour experiments and is monitored by the flow meter installed on the inlet pipe. Each scour case lasts 120 min. Then the water supply was stopped, and the measuring system started to collect the bed elevation data at each specific point around the deflectors. Detailed experimental information is shown in Table 1.

**Table 1:** Experimental conditions.

	1 Def	2 Def	3 Def
*B* (channel width)	0.2 m		
*H* (flow depth)	0.055 m		
*W* (Streamwise length of deflector)	0.06 m		
*L* (Spanwise length of deflector)	0.08 m		
$\upsilon$ (kinematic viscosity)	1.03E−06 m^2^/s		
*Q* (flow rate)	0.9 L/s		
*U*_*0*_ (incoming flow velocity)	0.082 m/s		
*Re* (*U*_*0*_*H*/$\upsilon$)	4.38E + 03		

Before the experiment began, water was slowly pumped from the storage flume into the recirculating flume using another small pump, after which the four main pumps gradually increased the flow rate from zero to the target flow rate within 1 min using a soft setup program. At the end of the experiment, the water in the recirculating flume was slowly pumped out, and the topography was measured using a small position sensitive infrared sensor (model GP2Y0A21) with a fast response time (0.1–0.5 s) and a small light beam radius (< 1 mm). The detection range of this infrared sensor is typically 10–30 cm with a measurement accuracy of 1 mm. Using a movement platform controlled by an Arduino board, the sensor moved quickly to the specified location at a speed of 10 cm/s.

## Numerical methodology

The three large eddy simulations were performed using the CFD code in ANSYS Fluent 15.0, which is based on finite volume discretization schemes. In the LES model, the large, anisotropic, and energetic scales of motion are reproduced by an unsteady and three-dimensional numerical integration of the Navier–Stokes (N–S) equations. The small, isotropic, and dissipative scales of motion are modeled in a SubGrid-Scale model (SGS). The scale separation is formally performed by applying a low-pass filter to the N-S equations. According to the implicit filter methodology, the filter width is proportional to the size of the computational cells that are used in the simulation. The flow equations obtained by filtering the 3D incompressible N–S equations are given by:

1 $$\frac{{\partial \overline{{u_{i} }} }}{{\partial x_{i} }} = 0$$

2 $$\frac{\partial }{\partial t}\left( {\rho \overline{{u_{i} }} } \right) + \frac{\partial }{{\partial x_{j} }}\left( {\rho \overline{{u_{i} }} \overline{{u_{j} }} } \right) = - \frac{{\partial \overline{p} }}{{\partial x_{i} }} + \frac{\partial }{{\partial x_{j} }}\left[ {\mu \left( {\frac{{\partial \overline{{u_{i} }} }}{{\partial x_{j} }} + \frac{{\partial \overline{{u_{j} }} }}{{\partial x_{i} }}} \right)} \right] - \frac{{\partial \tau_{ij} }}{{\partial x_{j} }}$$

where the overbar represents the resolved quantities, $u_{i} { }\left( {i = 1,2,3} \right)$ represents the velocity vector, $x_{i}$ (i = 1,2,3) represents the coordinate of the *i*-direction, *p* is the pressure, $\mu$ denotes the molecular viscosity, and $\tau_{ij}$ represents the sub-grid-scale stress (SGS), which reflects the influence of the small eddies modeled in an SGS model^28^ on large turbulence structures. The SGS stress was calculated using the eddy viscosity relationship:

3 $$\tau_{ij} = - \upsilon_{SGS} \left( {\frac{{\partial \overline{{u_{i} }} }}{{\partial x_{j} }} + \frac{{\partial \overline{{u_{j} }} }}{{\partial x_{i} }}} \right) + \frac{1}{3}\delta_{ij} \tau_{kk}$$

where $\delta_{ij} = 1$ when $i = j$; and $\upsilon_{SGS}$ = SGS viscosity, which is computed via an SGS model. The earliest SGS model is proposed by Smagorinsky^29^. This study utilized the Smagorinsky SGS model. In the near-wall region, because of the wall-resolved LES model needs much more computation resources, the Wener–Wengle wall-layer model was used in this paper. An analytical integration of the power-law near-wall velocity distribution resulted in the following expressions for the wall shear stress:

4 $$\left| {\tau_{w} } \right| = \left\{ {\begin{array}{*{20}l} {\frac{{2\mu \left| {u_{p} } \right|}}{\Delta z}\quad for\left| {u_{p} } \right| \le \frac{\mu }{2\rho \Delta z}^{{\frac{2}{A1 - B}}} } \hfill \\ {\rho \left[ {\frac{1 - B}{2}A^{{\frac{1 + B}{{1 - B}}}} \left( {\frac{\mu }{\rho \Delta Z}} \right)^{1 + B} + \frac{1 + B}{A}\left( {\frac{\mu }{\rho \Delta Z}} \right)^{B} \left| {u_{p} } \right|} \right]^{{\frac{2}{1 + B}}} \quad for\left| {u_{p} } \right| > \frac{\mu }{2\rho \Delta z}^{{\frac{2}{A1 - B}}} } \hfill \\ \end{array} } \right.$$

where *u*_p_ is the wall-parallel velocity, $\mu ,\rho$ denotes the molecular viscosity and density respectively, A = 8.3, B = 1/7 are the constants and $\Delta z$ the near-wall control volume length scale. By the advantage of the wall-layer model, the Y-plus value can be relaxed to 10, which is constrained to less than 1 in the wall-resolved model. The computational domain is meshed using about 3.9 million cells. The average $Y^{ + }$ is about 8, while the other two directions are about 2 times larger. The velocity profile at the inlet boundary is obtained from another independent straight channel numerical simulation. Further details and verification of the numerical model used in this study are described in Li et al.^30^.

**Figure 2:**
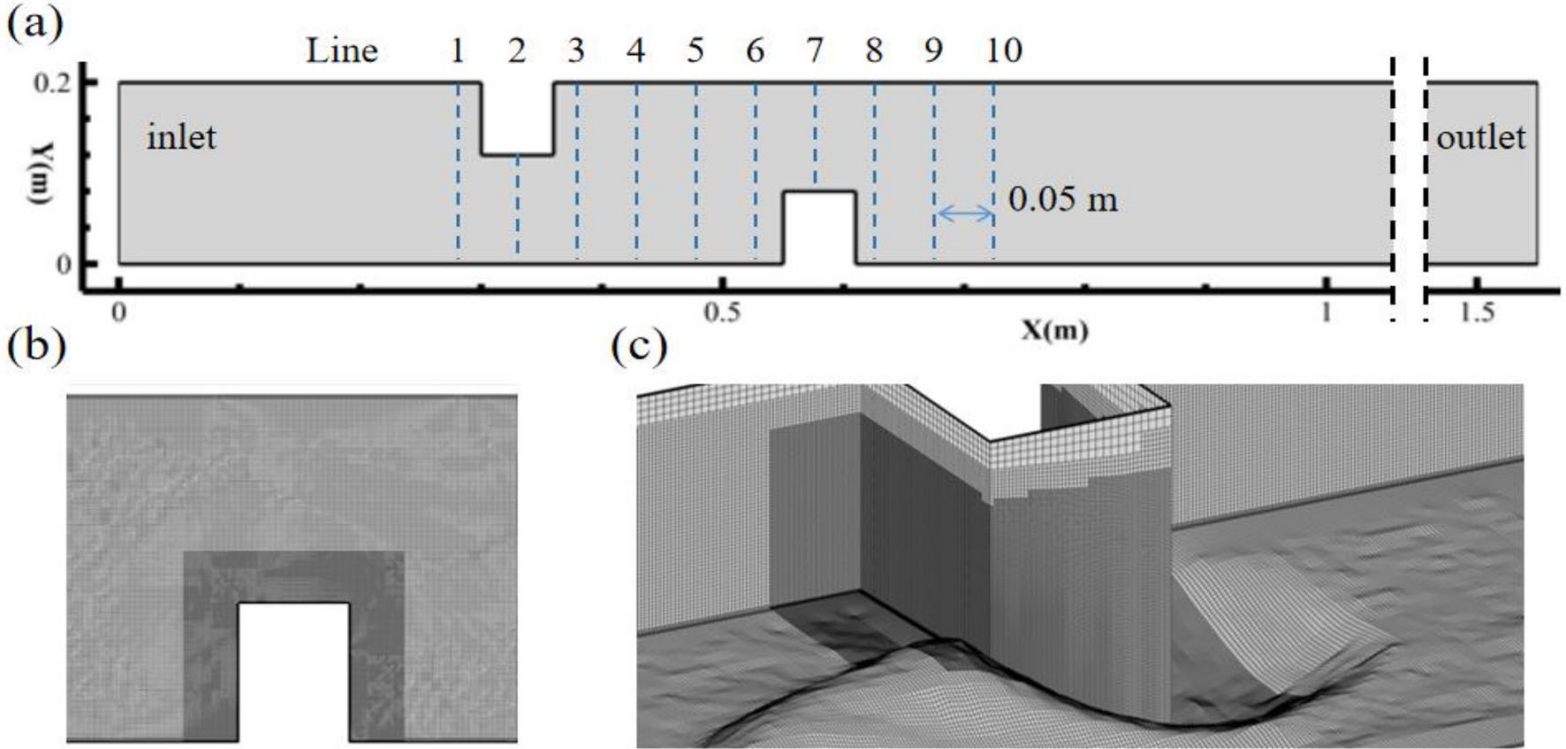
Position of the ten transverse lines on middle water depth plane.

The same scale numerical computational domain shown in Fig. 2a is established based on the above scouring experiments. The length of the numerical flume was taken as 1.6 m, and the scatter data of the bed elevation measured in the experiments were used to reconstruct the bottom boundary of the computational domain by interpolation (Fig. 2c shows an example of the reconstructed bed surface). The total number of grids is 6 million. The areas close to the wall were adapted, and the average Y+ of the first cells near the wall was approximately 0.8.

As mentioned above, both the experimental studies and numerical simulations demonstrate the existence of a complicated three-dimensional flow field near the spur dike, especially in the main scour hole. Thus, an accurate method for identifying the vortex system in an intricate flow field is essential for understanding the dynamic flow in the local scour problem. A vortex identification method called Rortex was proposed^31^, which is defined by the pure rotation of local fluid particles. Rortex showed good resistance to shear contamination^32,33^ compared with the Q criterion. Therefore, this study adopted the Rortex method to analyze the vortex systems.

## Results and discussion

### Topography

As shown in Fig. 2, similar topographic features were formed near the deflectors in both cases with the same flow rate ($Q = 0.9\;{\text{L/s}}$), including the main scour hole with a conical shape at the upstream corner of the deflector and strip deposition zone. The above scouring phenomenon was called basic scour in this paper. It is clear that the added deflector at the downstream zone had little influence on the basic scour of the first deflector at the upstream zone in Fig. 2b, except the maximum scouring depth of the main scour hole was slightly reduced. However, the scale of the main scour hole and deposition caused by the downstream deflector were significantly increased compared with the upstream deflector. The main reason attributed to this amplified effect was the accelerated flow induced by the upstream deflector, which made the downstream deflector face higher velocity and more turbulent flow than the upstream deflector. In addition, a new scouring pattern named elliptical scour hole appeared near the downstream deflector, which is depicted in Fig. 2b. Because the size of this elliptical scour hole was similar to the main scour hole, it can not be neglected.

An in-stream structure like the deflector will give the nearby flow field strong three-dimensional flow characteristics. The high-velocity fluid, the down flow, and the vortices induced by the structure will cause the movement of the sediment particles to form scouring. In addition to the above-mentioned flow structures, the fluid could be guided to a meandering flow with alternating deflectors. The analysis of the meandering flow structure in the following sections could help to understand the elliptical scour hole brought by the alternating layout.

### Meandering flow

**Figure 3:**
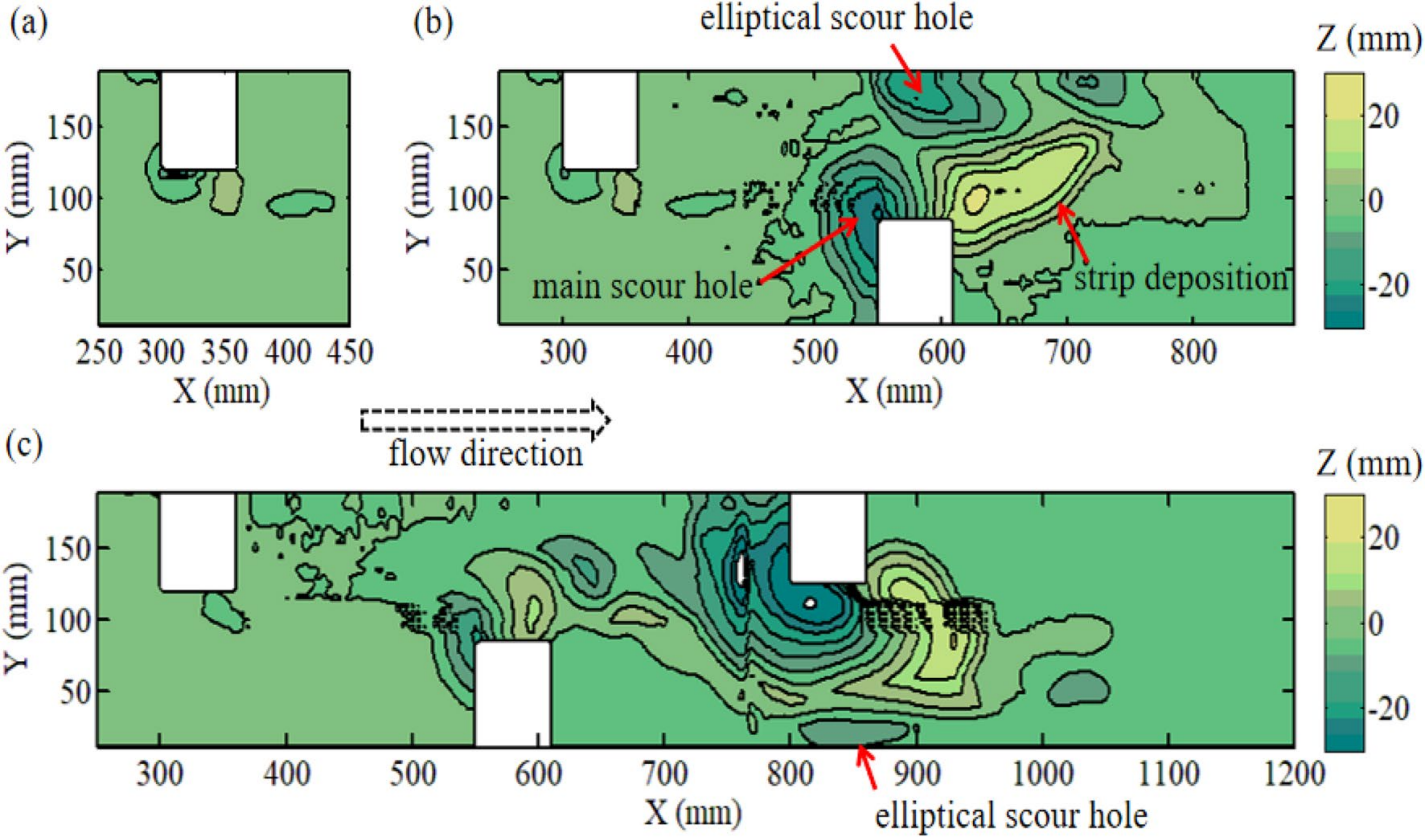
Topography of scoured bed: (**a**) single deflector; (**b**) double deflectors.

Due to the contraction effect of the deflector on the cross-section of the channel, the single deflector accelerates the main flow once, while the double deflector could accelerate the main flow twice in series if the spacing of double deflectors is controlled reasonably. In this study, the second flow acceleration region produced by the downstream deflector was notable and had a higher speed compared to the first accelerated flow. The velocity magnitude distribution on three different horizontal planes (z = 0.05, 0.0275, 0.005 m respectively) in Fig. 3 shows that the leg of the second acceleration flow (AF2) had obvious jet flow characteristics when the space of the two deflectors was 0.25 m. At three planes, the jet flow belonging to AF2 was all close to the side wall of the channel with a relatively narrow lateral range (< 0.08 m) and a longer longitudinal distance (> 0.8 m). At the middle water depth plane, high velocity area (> 0.24 m/s) was larger than the rest planes.

In addition, there is a process in which the flow was deflected from one side wall to the other between the two acceleration flows. The first acceleration flow (AF1) produced by the upstream deflector was blocked by the downstream deflector during its forward movement. Then the velocity component of the accelerated flow was reversed in the transverse direction (Y). And the fluid approached the side wall at Y = 0.2 m. The above process of flow deflection formed a simple meandering flow structure, was shown by the dotted line in Fig. 3b.

**Figure 4:**
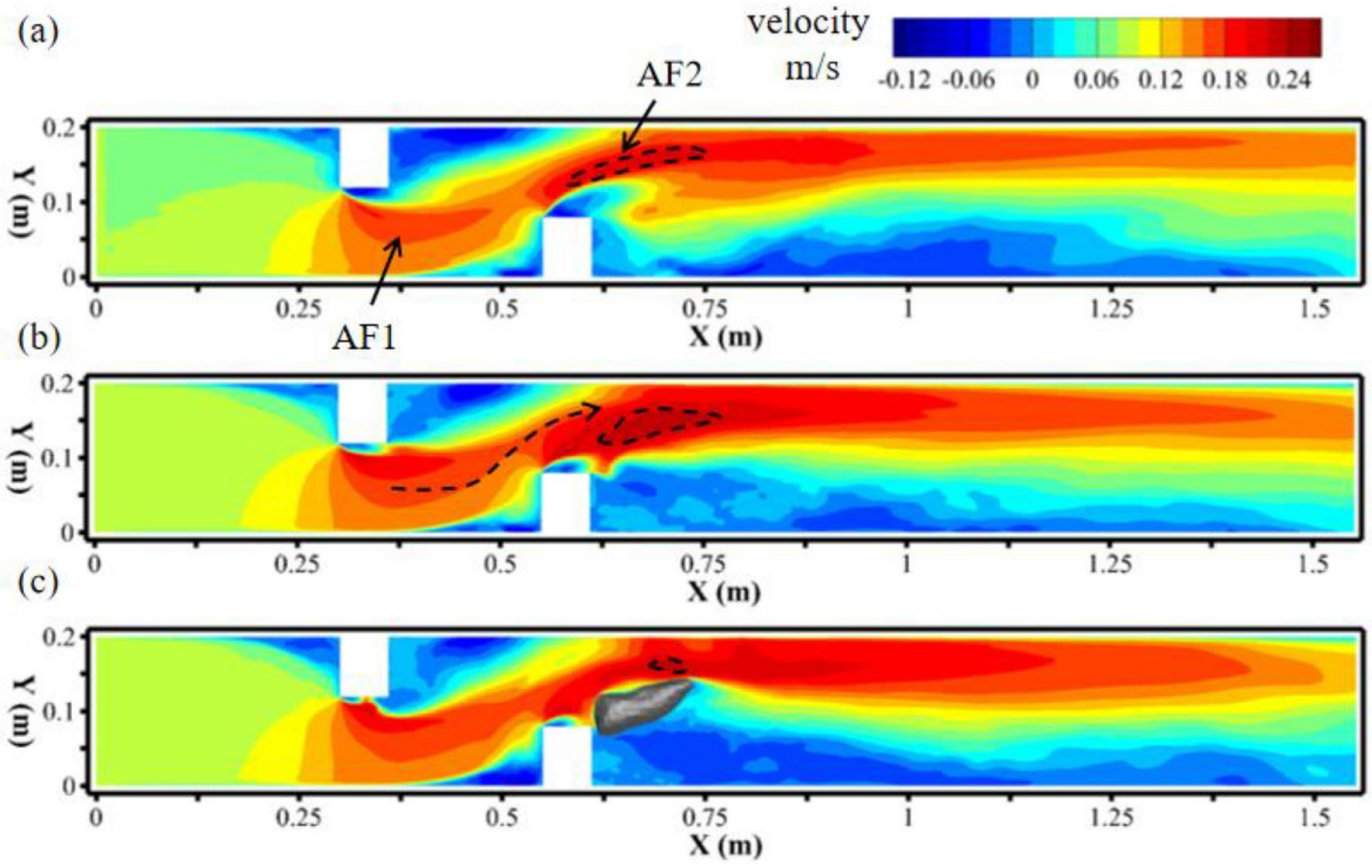
The time-average velocity on three different horizontal depth plane obtained from numerical results: (**a**) *z* = 0.05 m; (**b**) *z* = 0.0275 m; (**c**) *z* = 0.005 m.

In order to analyze the detail of the acceleration and deflection process induced by two alternating deflectors, ten transverse lines were chosen at equal intervals (0.05 m) along the direction of the flow in the medium water depth plane as shown in Fig. 4. The longitudinal velocity profiles (u) on these lines represent the acceleration effect of the deflectors on the main flow, and the transverse velocity profiles (v) represent the deflection effect of the deflectors on the main flow.

**Figure 5:**
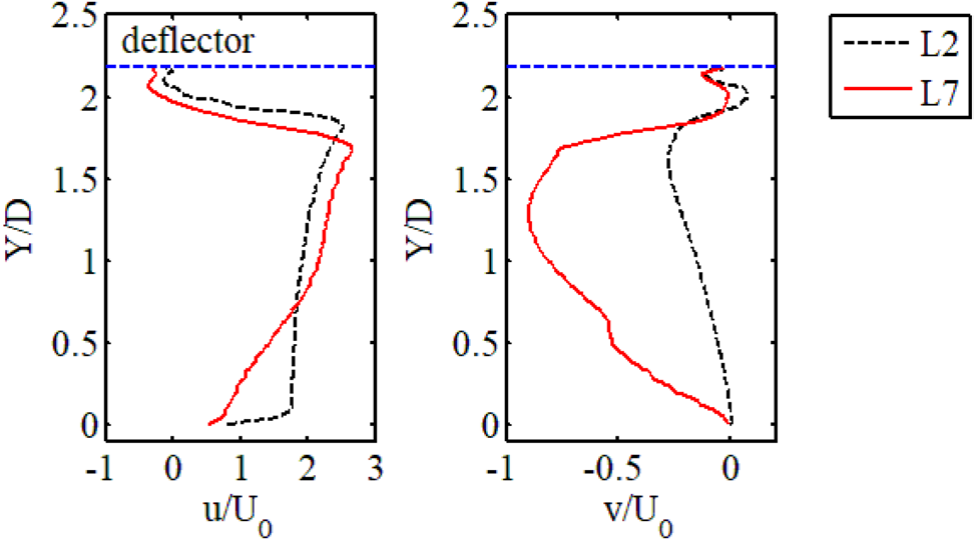
Comparison of longitudinal and lateral velocity between line 2 and line 7.

Since line 2 and line 7 were located in the middle of the deflector, the velocity profiles of these two lines were used to compare the difference in the influence of the two deflectors on the main flow. Because of the extension direction of the two deflectors was opposite, line 7 in Fig. 5 was rotated by 180 degrees for the convenience of comparison. The longitudinal velocity (u) profile shows that the acceleration effects of the two deflectors on the main flow were similar. Except that the maximum velocity generated by the downstream deflector was slightly higher, and the accelerated region was narrower. The main reason was that the longitudinal velocity (u) decayed rapidly near Y = 0 due to the influence of the recirculation zone generated by the upstream deflector. The lateral velocity profiles show that the two deflectors deflected the fluid in opposite directions, and the downstream deflector had a stronger deflecting effect.

**Figure 6:**
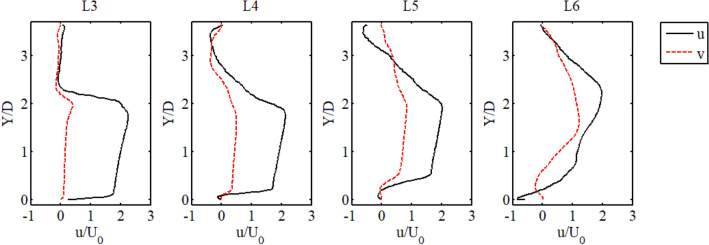
Velocity profile of line 3 to line 6.

The deflection process of the main flow was located between the two deflectors, and the velocity profiles of line 3 to line 6 are shown in Fig. 6. At X = 0.38 m (Line 3), the main flow was concentrated in the range from Y = 0 m to Y = 0.12 m. The dimensionless longitudinal velocity (u/U_0_) in most of this region was higher than 1, indicating an acceleration state. The transverse velocity was small, indicating that the main flow had not been deflected. At X = 0.43 m (Line 4), the longitudinal velocity appeared to have a negative value near Y = 0 and Y = 0.2, which means that this section had entered two recirculation zones caused by the two deflectors. At the same time, the transverse velocity was slightly increased but still small, so the longitudinal velocity profile maintained the previous distribution shape at X = 0.38 m. At X = 0.48 m the main flow began to be deflected due to the transverse velocity continuing to increase. Therefore, the position of the AF1 started to move towards Y = 0.2 m. At X = 0.53 m, the transverse velocity was significantly enhanced, and the fluid was strongly deflected. The shape of the AF1 was completely changed while moving further to Y = 0.2 m. In summary, the flow deflection effect represented by the transverse velocity has a great influence on the distribution and shape of the acceleration area, so the downstream deflector has a great influence on the AF1, which could induce a simple meandering flow pattern.

**Figure 7:**
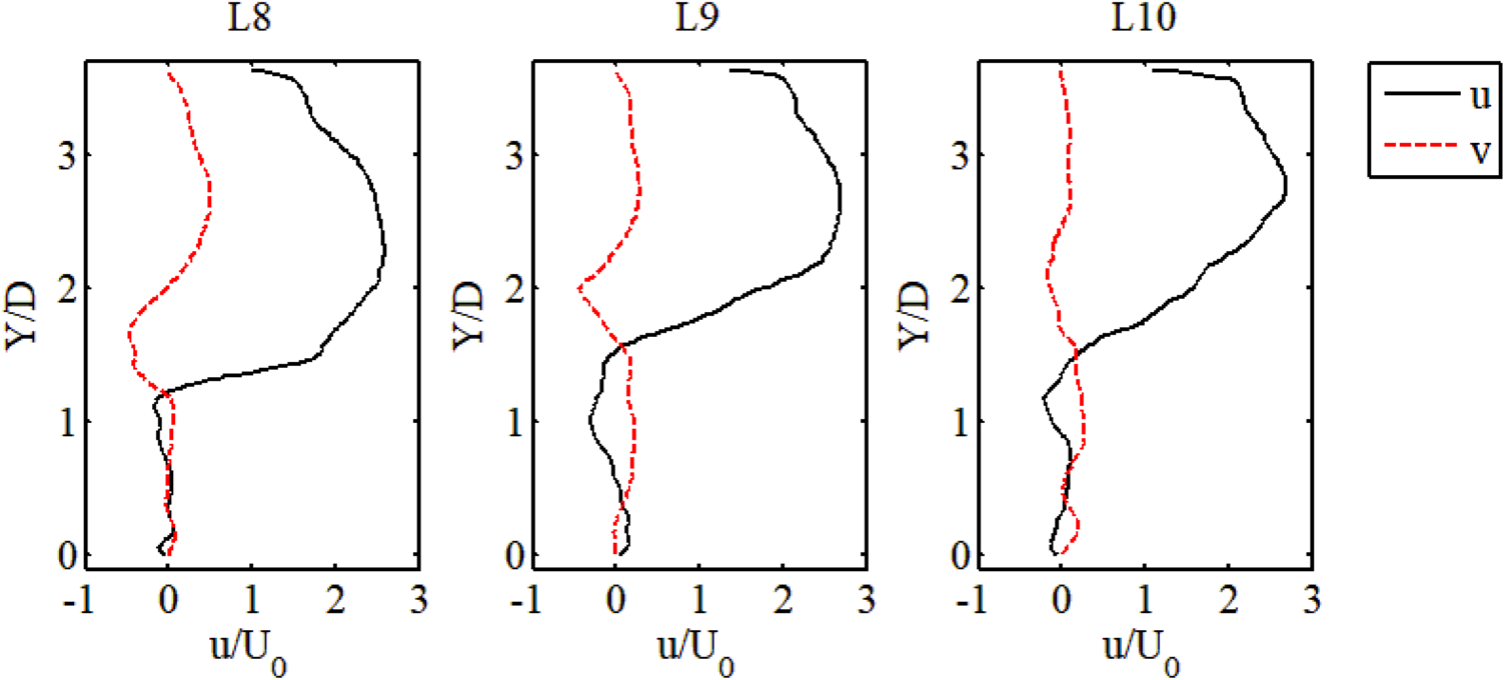
Velocity profile of line 8 to line 10.

The meandering flow was accelerated again after the deflection and was located in the downstream area of the channel. At X = 0.63 m and X = 0.68 m in Fig. 7, the transverse velocity had negative values around Y = 0.1 m. This position was just at the boundary between the high-speed and low-speed zones, so it can be seen that the transverse range of the high-speed zone (i.e. AF2) further decreased as the X distance increased. This explains why the distribution of AF2 can be close to the channel wall while having a large aspect ratio.

**Figure 8:**
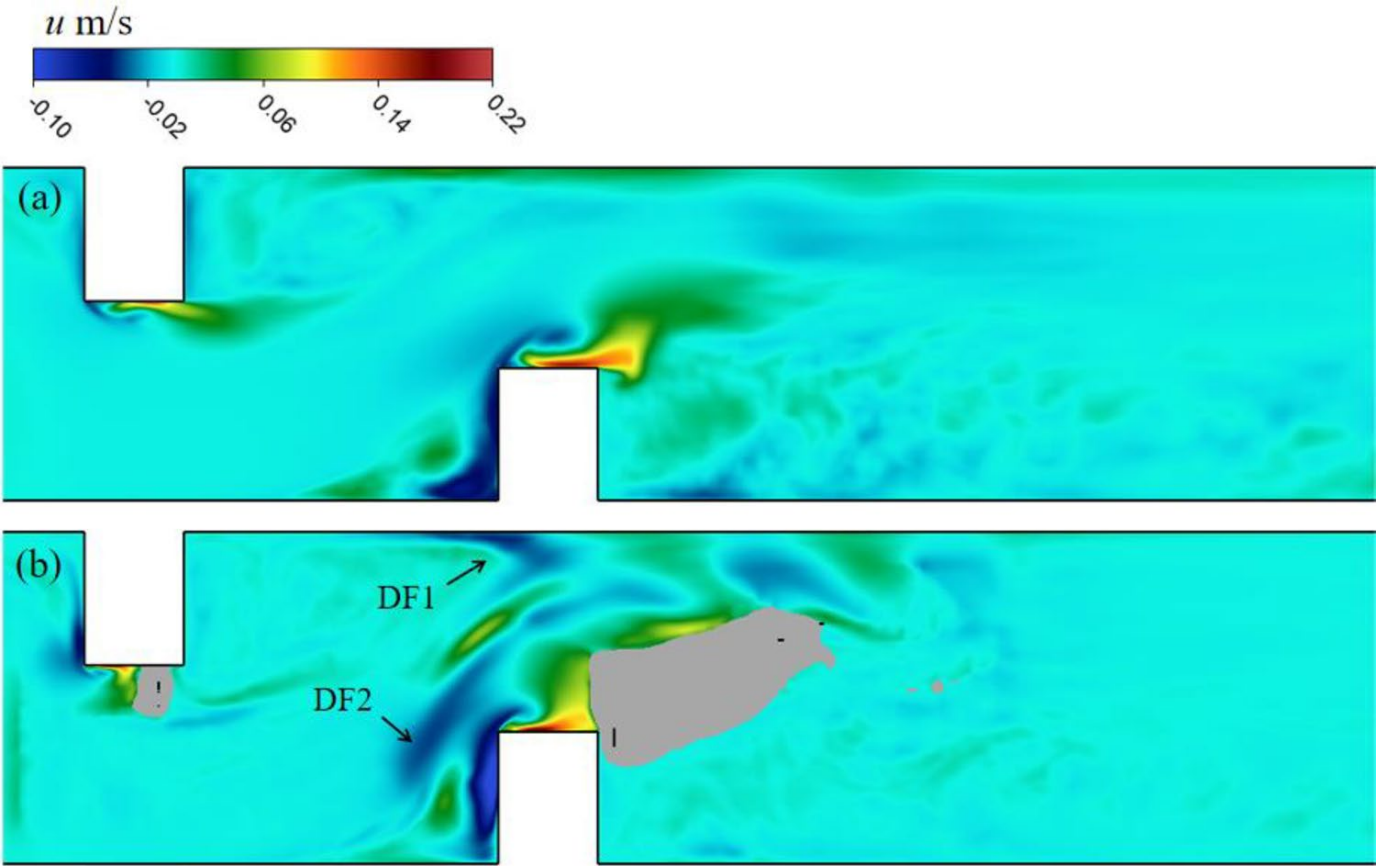
Distribution of vertical velocity on (**a**) middle water depth plane z = 0.0275 m and (**b**) near the bottom plane z = 0.005 m.

In addition to flow acceleration (AF1 and AF2), the blocking effect of the deflectors on the open channel flow also produced a downward flow, especially in the upstream area of the downstream deflector. Figure 8 shows the vertical velocity distributions (negative value represents downward flow) on two horizontal planes, including the middle water depth plane and near bed plane. The results show that the downward flow areas were mainly concentrated near the upstream surfaces of the two deflectors on the middle water depth plane, and there were two additional areas of downward flow on the near bed plane (black arrows in Fig. 8b). Downward flow area 1 (DF1), which was close to the channel wall, was located on the path of the deflected flow. DF1 was generated by the accelerating flow in AF1 being forced to turn by the downstream deflector and hit the channel wall (Fig. 8a). Another downward flow area (DF2) was located in the scour hole near the corner of the downstream deflector. It was presumed that DF2 was related to the flow moving into the scour hole as a result of the blockage of the downstream deflector. The downward flow could affect the scour process of the bed, especially the elliptical scour hole located under the DF1 area, which was the scour pattern caused by the meandering flow. Thus, it was important to analyze the flow structure near the elliptical scour hole.

**Figure 9:**
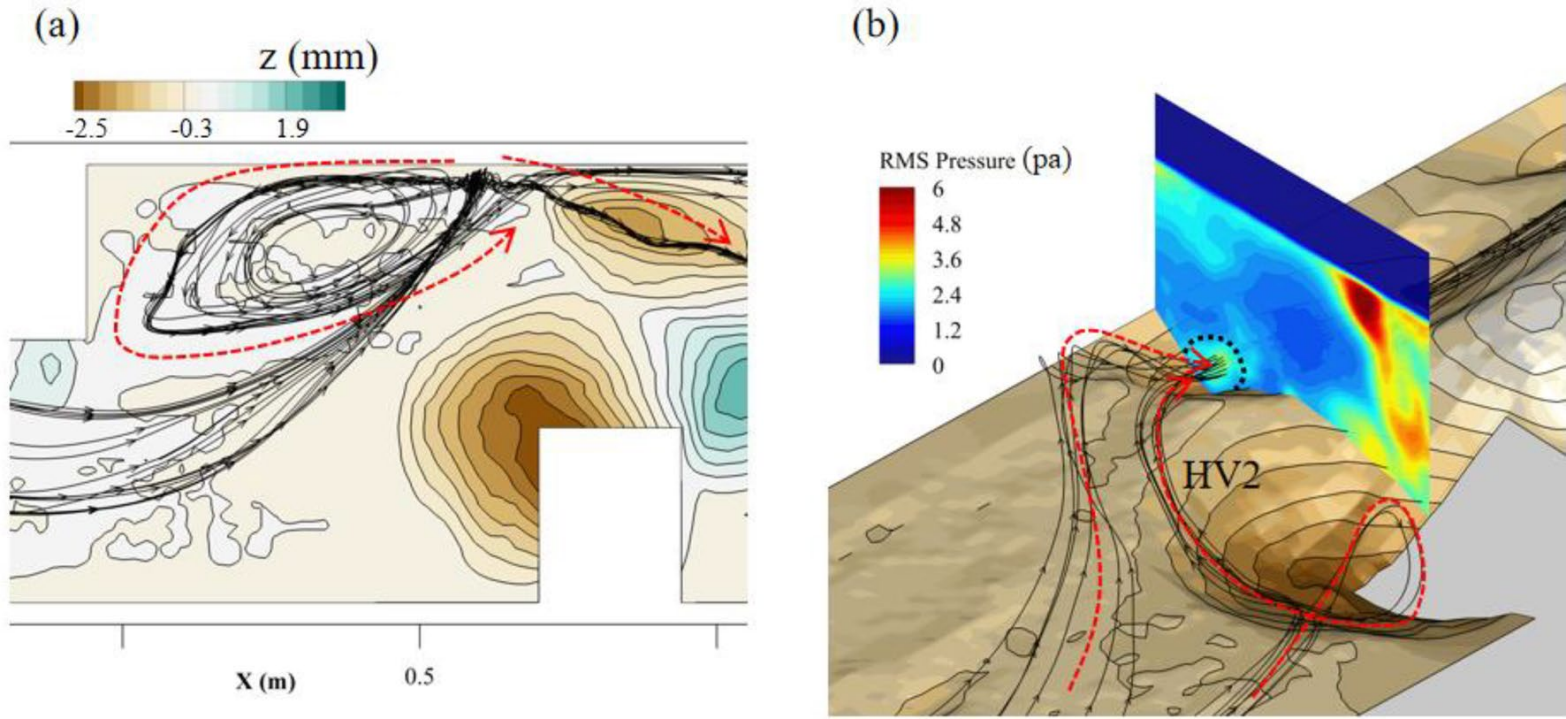
Flow structure generated by downward flow nearby the elliptical scour hole: (**a**) top view; (**b**) side view.

Three-dimensional streamlines were used to visualize the flow structure through the DF1 area as shown in Fig. 9a. It can be found that there are two flow paths with opposite movement directions. One entered the recirculation zone induced by the upstream deflector. And the other one moved into the elliptical scour hole with a spiral flow path (Fig. 9b). The root means square (RMS) of pressure on the cross section (Fig. 9b) indicated that a pressure fluctuation was generated inside this spiral flow structure. Because the pressure fluctuation area was close to the bed surface, it could affect the development of the elliptical scour hole. In addition, the streamlines in Fig. 9b also show that the spiral flow structure in the elliptical scour hole was composed of not only the fluid fed by DF1, but also the fluid coming from the second horseshoe vortex (HV2) generated by the downstream deflector.

**Figure 10:**
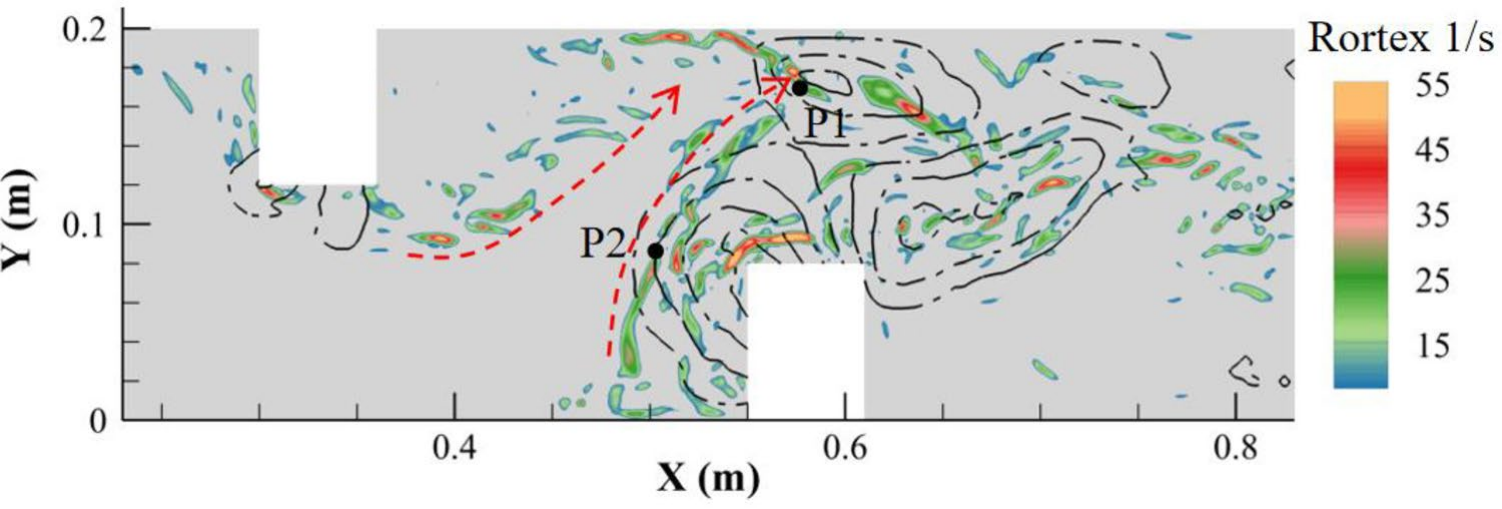
Distribution of Rortex value on z = 0.005 m plane in double deflectors case.

**Figure 11:**
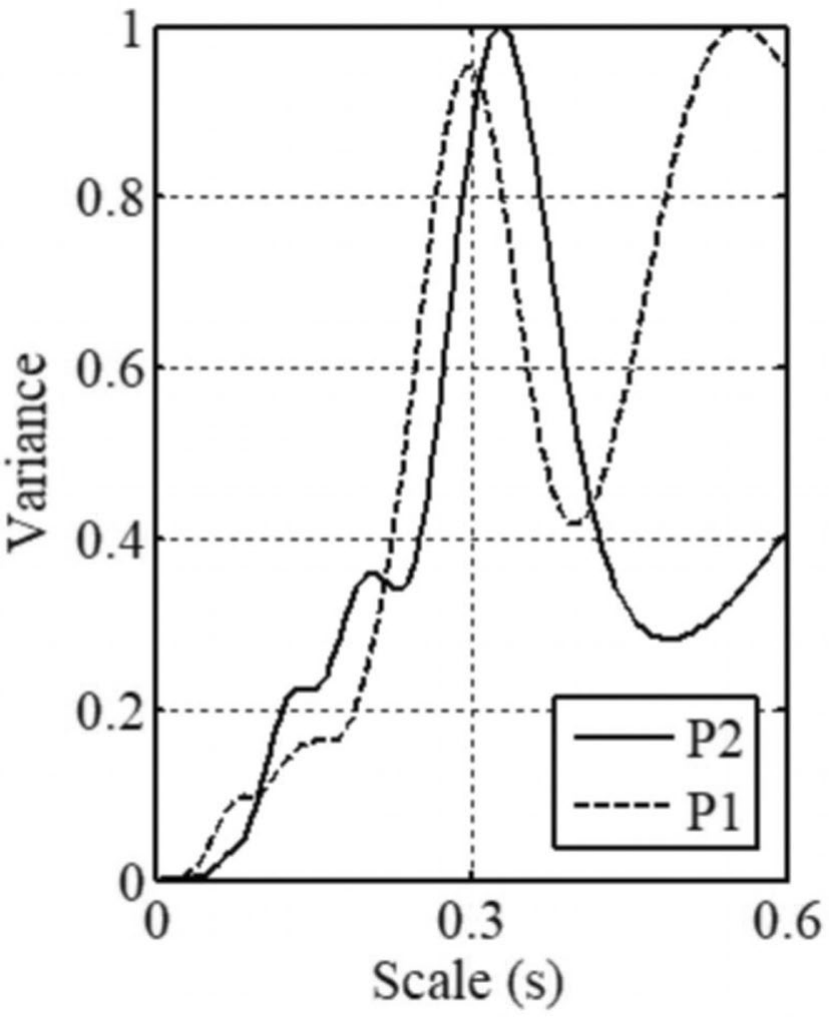
Wavelet analysis of Rortex value varied with time at point1 and point2.

The instantaneous distribution of the eddies on the near bed plane in Fig. 10 also depicted the phenomenon that the wake vortices of HV2 moved toward the elliptical scour hole after shedding. These wake vortices had significant energy because the intensity and size of HV2 were the highest in the near-bed area of the entire channel. Therefore, the spiral flow structure inside the elliptical scour hole should be greatly affected by HV2. Two monitoring points were set at the center of the elliptical scour hole (P1) and the tail of HV2 (P2), respectively. The wavelet method was used to analyze the Rortex value, which varied with time, as shown in Fig. 11. It was found that the two points had similar minimum main periods (approximately 0.3s), which confirmed the influence of the area of HV2 on the flow structure in the elliptical scour hole. In conclusion, both DF1 and HV2 could affect the development of the elliptical scour hole.

### Influence of downstream deflector

**Figure 12:**
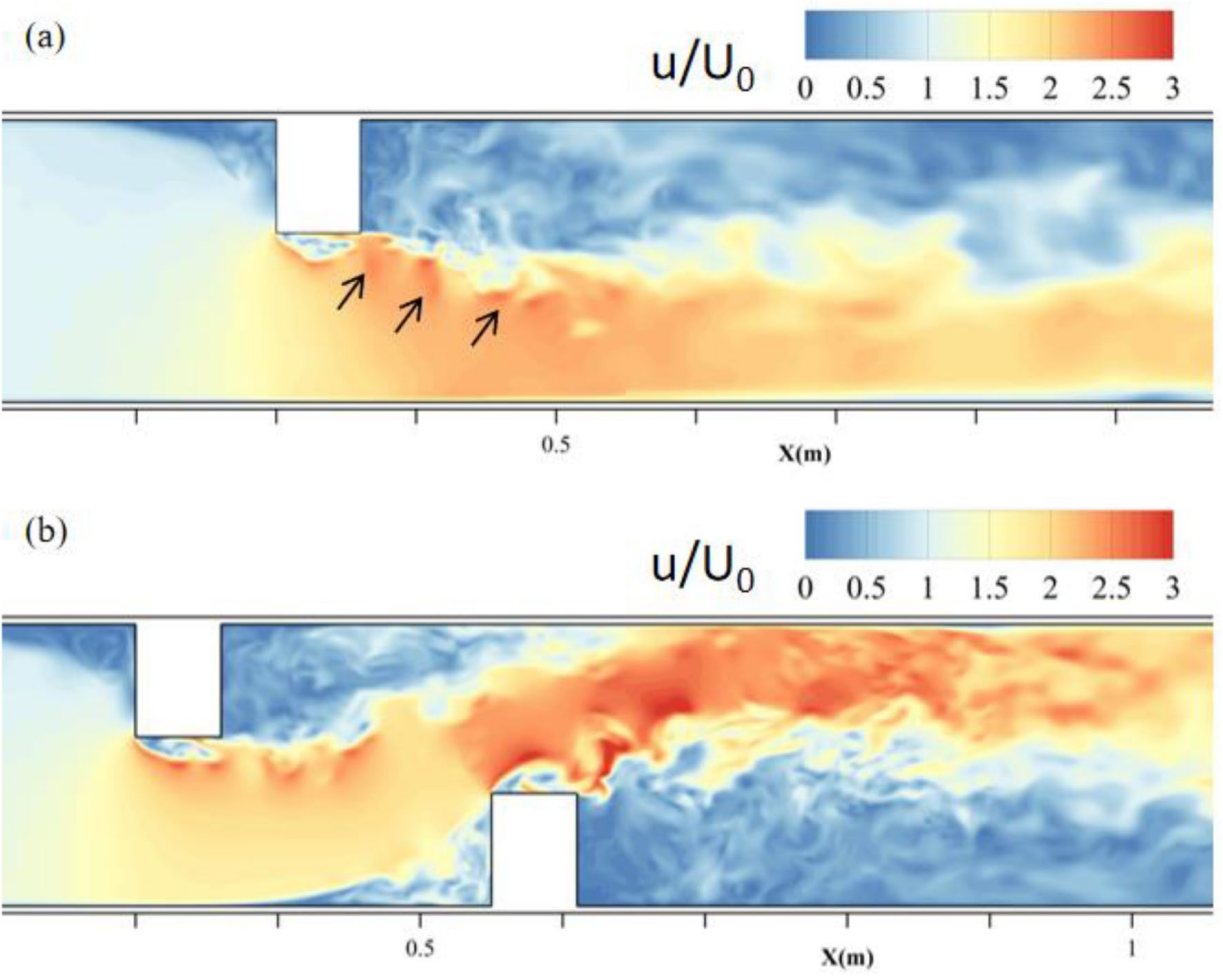
Velocity distribution on middle water depth plane with (**a**) single deflector and (**b**) double deflectors.

The numerical results of a single deflector with a scoured bed and a double deflector with a scoured bed were compared to study the influence of the downstream deflector on the flow field at the upstream area. In the case of a single deflector, the upstream corner of the deflector generated a shear stress layer with a random disturbance inside. This disturbance propagated downstream without restriction, along with the shear stress layer, and induced a series of local high-speed flow areas, as shown by the black arrows in Fig. 12a. However, the propagation of the accelerated flow was restricted in the case of double deflector as shown in Fig. 12b. And the phenomenon of the high-speed patch disappeared within AF2 and was replaced by a higher velocity zone with a narrower scope.

**Figure 13:**
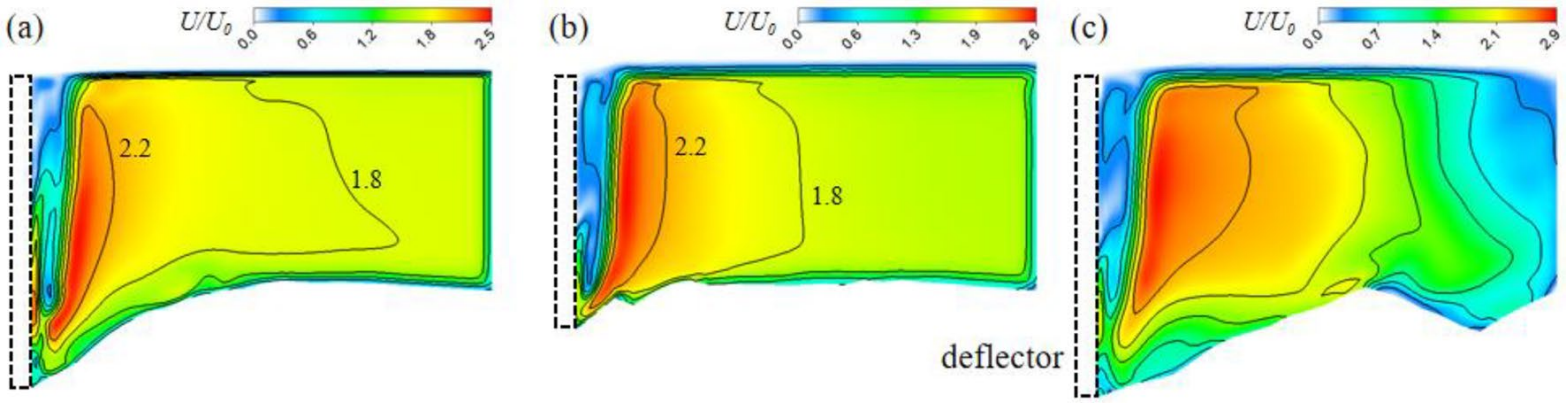
Velocity contours on cross-section plane: (**a**) single deflector *x *= 0.31 m; (**b**) double deflector *x* = 0.31 m; (**c**) double deflector *x *= 0.56 m.

Figure 13 depicts the comparison of the distribution of the velocity magnitude on the cross-sectional plane. It shows that the maximum velocity in the shear stress layer increased after the downstream deflector was added. At the same time, the density of the counter lines of velocity increased in the shear stress layer. In other words, the accelerated flow was more concentrated in the double deflector case.

**Figure 14:**
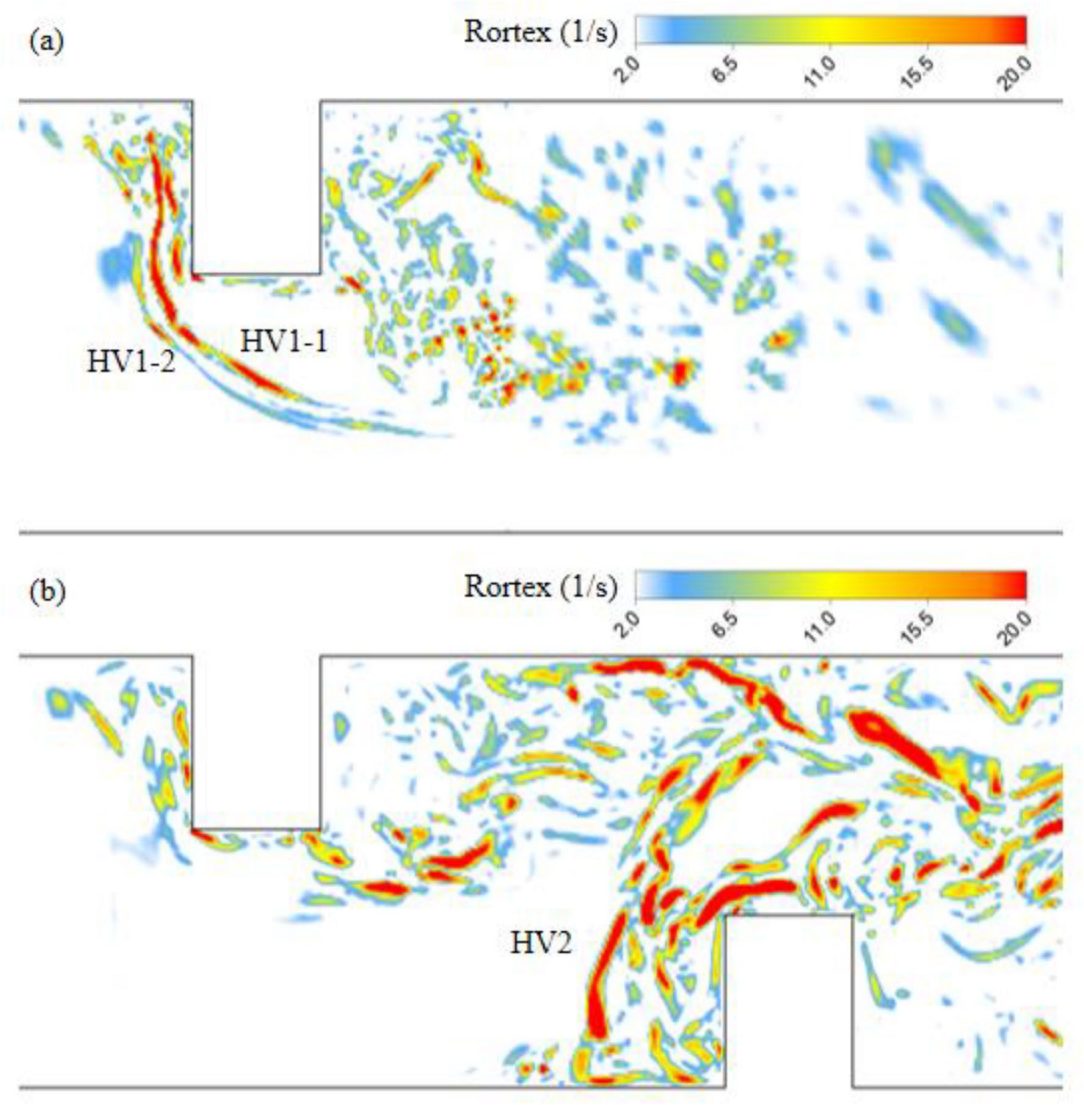
Rortex distribution on near bed plane, (**a**) single deflector with scoured bed (**b**) double deflectors with scoured bed.

**Figure 15:**
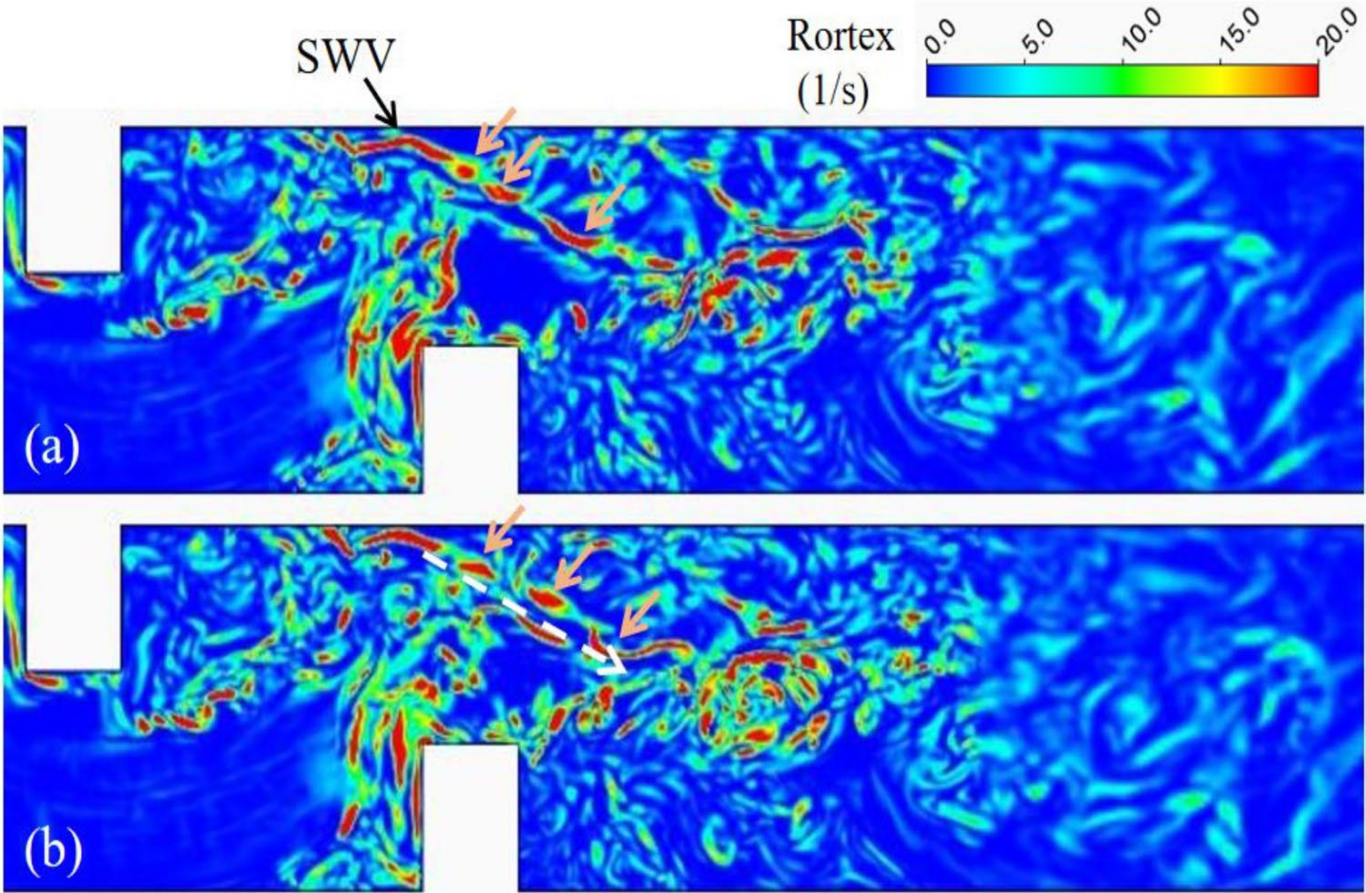
Vortex shedding in the meandering flow on middle depth plane of double deflector case.

**Figure 16:**
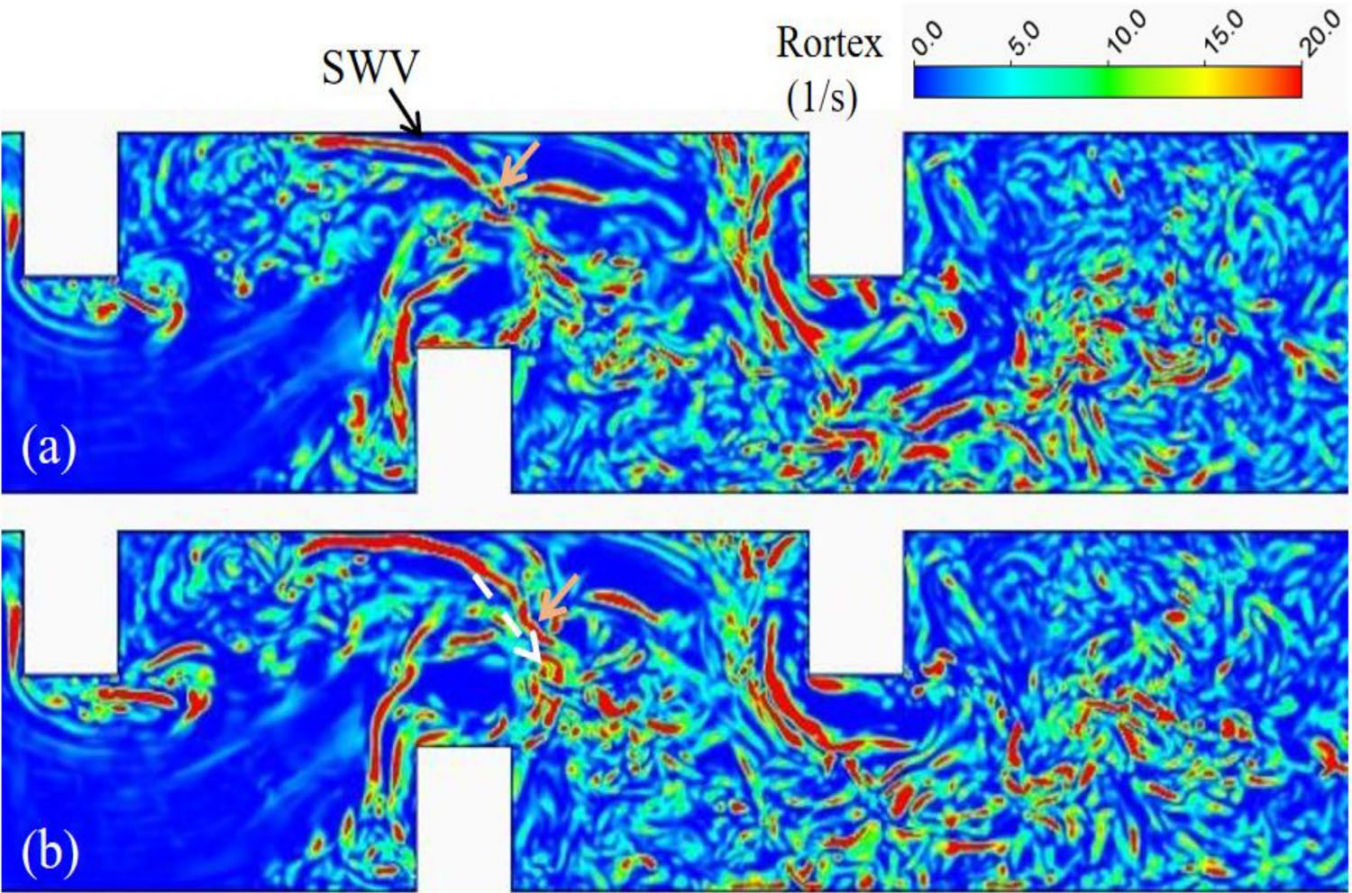
Vortex shedding in the meandering flow on middle depth plane of three deflectors case.

The downstream deflector also affected the horseshoe vortex system (HV1) of the upstream deflector. The disappearance of HV1-1 and HV1-2 in the upstream region is evident in Fig. 14, with the shedding wake vortices becoming more prominent as a result of the increased shear stress layer. A large and continuous side wall vortex (SWV) is observed in the curved flow generated by both two and three deflectors located at the opposite bank of the second deflector. This SWV exhibits continuous tail vortex shedding, moving towards the channel center, illustrated by the orange arrows in Figs. 15 and 16. Under the double deflector condition, the SWV is precisely situated within the elliptical scour pit region. The motion of the tail vortex, depicted by the white arrows in Fig. 15b, aligns with the extension of the elliptical scour crater, indicating that the tail vortex motion influences the evolution of the scour hole and modifies its morphology.

**Figure 17:**
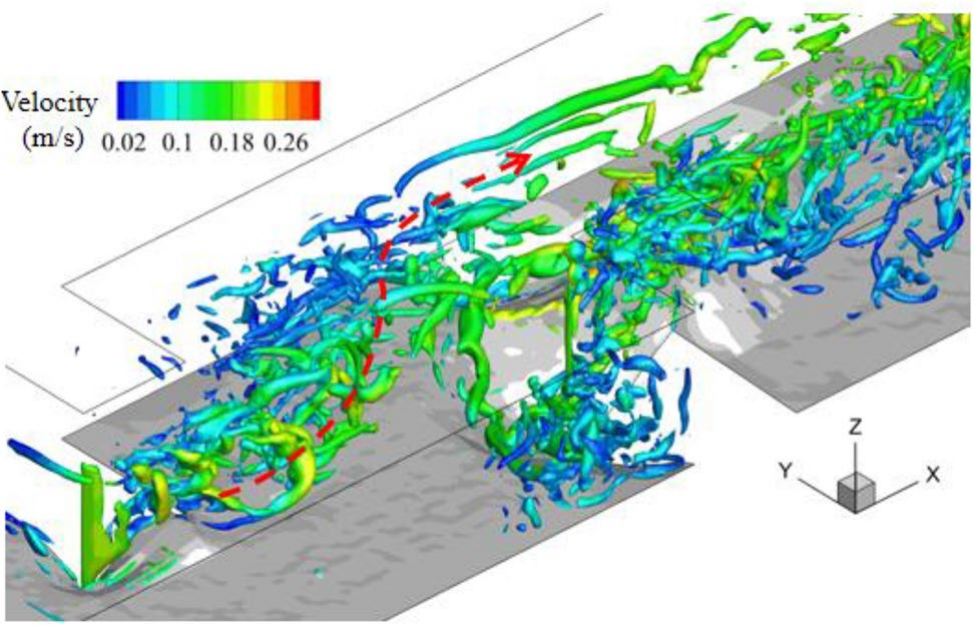
Vortices showed by Rortex in meandering flow.

In both cases, most eddies were generated from the upstream corner of each deflector and then moved to the downstream region. However, the transportation path of the vortices generated by the upstream deflector is obviously affected by the meandering flow as shown in Fig. 17. After passing through the first deflector, the wake eddies quickly been deflected and moved forward to the bank. Then part of these eddies moved along with the side wall. And the elliptical scour hole was located just below this path.

## Conclusion

After installing alternating deflectors in a straight channel, a meandering flow pattern was formed between the double deflectors through two flow acceleration processes and one deflection process. At the same time, a high-speed jet flow zone close to the channel wall was formed in the downstream area for a long distance.

The accelerated flow generated by the upstream deflector was deflected by the downstream deflector and hit the channel wall at a certain angle, which formed a strong downward flow region. This downward flow region contained two fluid motions in opposite directions: (a) one entered the recirculation zone and (b) the other one moved toward the center of the channel. The latter moved into an elliptical scour hole in a spiral path and formed a spiral flow structure. This spiral flow structure was affected by the second horseshoe vortex (HV2) because of the shedding wake vortices from the leg of HV2. The downstream deflector also had some influence on the upstream region, including the concentration of the upstream fluid acceleration region and the weakening of the horseshoe vortex system.

The current numerical results were based on one hydraulic condition, and it was used to speculate that the development of an elliptical scour hole is related to the spiral flow structure. However, the flow patterns (spiral flow, eddies, etc.) depend on the longitudinal distance and the lateral spacing between the two deflectors, and the present results could not prove that the spiral flow structure was the main factor causing the elliptical scour hole. Further experimental research would be an effective way to investigate the scouring process of the elliptical scour hole.

## Data Availability

All data, models, or codes that support the findings of this study are available from the corresponding author upon reasonable request.
